# Renal Solid Mass as a Rare Presentation of Wagener's Granulomatosis: A Case Report

**DOI:** 10.1155/2012/793014

**Published:** 2012-08-05

**Authors:** Mehrdad Mohammadi Sichani, Mazaher Hadi, Ardeshir Talebi, Pooyan Khalighinejad

**Affiliations:** ^1^Department of Urology, Isfahan University of Medical Sciences, Isfahan 81746-75731, Iran; ^2^Department of Pathology, Isfahan University of Medical Sciences, Isfahan 81746-75731, Iran; ^3^School of Medicine, Isfahan University of Medical Sciences, Isfahan 81746-75731, Iran

## Abstract

Wagener's granulomatosis (WG) is a rheumatologic disease with unknown etiology which renal and pulmonary involvement is commonly seen. Renal involvement in Wagener's granulomatosis represents as a segmental necrotizing glomerulonephritis which is not visible with imaging techniques and usually presents with proteinuria, microhematuria, and hypertension. A rare presentation of the disease is a renal mass which can be mistaken as renal tumors, abscess, or lymphoma. We report a 22-year-old female with flank pain and fever who was admitted in our hospital. The patient underwent renal tumor biopsy and diagnosed with Wagener's granulomatosis in pathologic staining. The aim of this work is introduction of Wagener's granulomatosis as a differential diagnosis of renal tumors, to prevent unnecessary interventions and delayed treatment.

## 1. Introduction 

Renal involvement in Wagener's granulomatosis represents as a segmental necrotizing glomerulonephritis which is not visible with imaging techniques and usually presents with proteinuria, microhematuria, and hypertension [1]. A sparse manifestation of this disease is renal mass that may be mistaken by renal tumors, abscess, or lymphomas [1]. We report a case that was referred to us with a fever and flank pain which was suspected to renal infection.

## 2. Case Report

A 22-year-old female presented to our centre with ten-day high fever of unknown origin. She was well and healthy until 6 months ago when she developed generalized arthralgia without obvious arthritis. She was seen by a local doctor and treated for symptomatic management. Her past medical history reveals a total mastoidectomy secondary to chronic otitis. Nonpurulent otorrhea already existed after surgery. 

**Figure 1:**
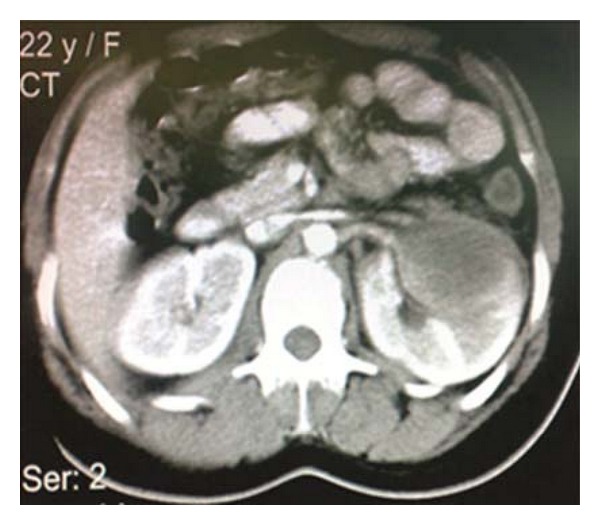
CT scan shows hypodense renal mass with ring enhancement.

On physical examination, she had generalized weakness, arthralgia, nonpurulent otorrhea, partial deafness of left ear, and tenderness at the costovertebral angles. The oral temperature was 39°C. The rest of her physical exam was normal. The blood tests revealed anemia (Hb: 8.2 gr/L), elevated white cell count (WBC: 23700/mm^3^), elevated sedimentation rate (ESR: 136), and CRP. The urinalysis showed microscopic hematuria and proteinuria. The renal functions tests, serum electrolytes, and coagulation tests were in normal ranges for her age. The ultrasound study of her abdomen and pelvis showed a 6 cm heterogenic mass in the upper and lower pole of the left kidney. The patient underwent abdominopelvic spiral CT scanning with IV contrast which revealed a hypodense mass at the left kidney suggesting of a renal abscess, tumors or less likely infiltrative lesions like lymphomas (Figure 1).

**Figure 2:**
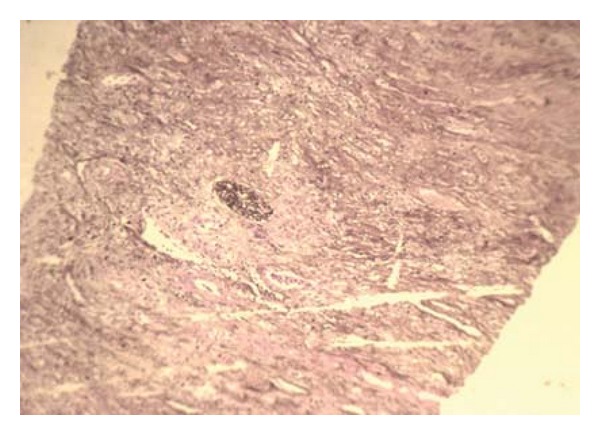
Photomicrograph (Jones staining ×40) showing a normal glomerulus.

**Figure 3:**
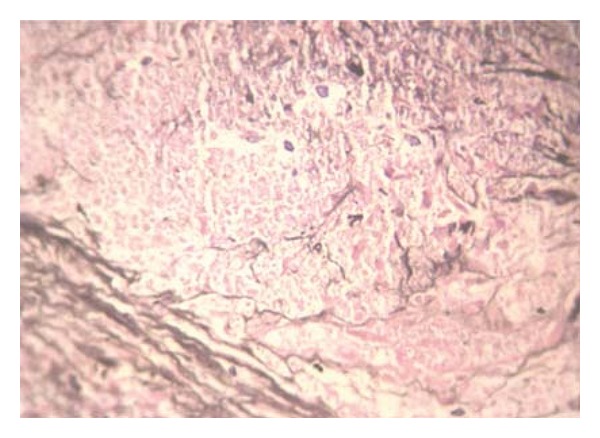
Photomicrograph (Jones staining ×400) showing granuloma and necrosis.

**Figure 4:**
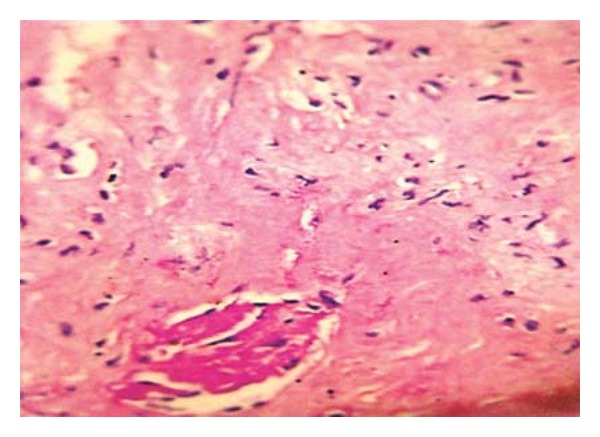
Photomicrograph (Jones staining ×400) showing sclerosis of a glomerulus.

The initial diagnosis was a renal abscess considering persistent fever and flank pain for 48 hours despite broad spectrum antibiotic therapy. Percutaneous ultrasound guided drainage of the presumed renal abscess was done, however, the aspirated fluid was clear without any pus or debris. She then underwent a core needle biopsy. The patient tolerated the procedure well and there was no immediate complication. Rheumatologic screening tests were also sent off. The ANCA test was positive and pathologic study of the tissue sample with H&E, Masson, and Jones revealed sclerosis, granulomatosis inflammation, multinucleated giant cells, vasculitis, and severe fibrinoid necrosis without any malignant cell suggestive of Wagener's granulomatosis (Figures 2, 3, and 4). Unfortunately, a day after the biopsy, the patient died due to massive hemoptysis, and cardiopulmonary arrest. 

## 3. Discussion

Although renal involvement is seen in 80% of patients with Wagener's granulomatosis, 20% of patients have renal entanglement at time of diagnosis [1]. Renal biopsy findings include rapidly progressive glomerulonephritis with active urine sediment [2]. The first case of renal mass in Wagener's granulomatosis was reported in 1978. After those only 12 cases with the same presentation were reported [1]. Ten of them had a unilateral renal mass which Wegener's disease was diagnosed by pathologic examinations [1]. Only in 2 of these cases, renal mass was the first presentation of the disease [3, 4]. In all reported cases, pathological examination of renal mass revealed granulomatosis [5]. In Roussou et al.'s study in 2008 [6], an association between Wagener's granulomatosis and renal cancer was reported and, therefore, they suggested to perform a biopsy in all patient with Wagener's granulomatosis patients and a renal mass.

Our patient presented with a history of ten day fever and flank pain. Arthralgia and otorrhea were ignored because of the severity of flank pain and a high-grade fever. 

Renal mass is a rare presentation of Wagener's granulomatosis and its clinical and radiologic manifestations are similar to a renal abscess, tumor, or an inflammatory process. Taking a meticulous history and physical examination and high clinical suspicion is of great importance to prevent unnecessary interventions and delay treatment. This patient was in uncontrolled phase of the disease which every surgical intervention was life threatening for her. If we paid attention to her history, we found that she had a typical history of a chronic systemic disease, so an appropriate medication could control the acute phase of the disease and save her life. 

In the previous studies, patients treated with immunosuppressive and corticosteroid showed improvement of their clinical symptoms including subsiding of the renal mass. Our patient died during the diagnostic evaluations due to a sudden onset massive hemoptysis.

## 4. Conclusion 

A renal mass is a rare presentation of Wegener's granulomatosis. High grade of suspicion and detailed history and physical examination are required for early diagnosis and treatment.
